# Non breathing-related sleep fragmentation and imaging markers in patients with atherosclerotic cerebral small vessel disease (CSVD): a cross-sectional case-control study

**DOI:** 10.1186/s12883-020-01647-x

**Published:** 2020-03-17

**Authors:** Jihui Wang, Xiaodong Chen, Jinchi Liao, Li Zhou, Hongying Han, Jiong Tao, Zhengqi Lu

**Affiliations:** 1 Department of Psychiatry, the Third Affiliated Hospital, Sun Yat-sen University, Guangzhou, 510630 China; 2 Department of Neurology, the Third Affiliated Hospital, Sun Yat-sen University, No. 60, Tianhe Road, Tianhe District, Guangzhou, 510630 China; 3 Department of Rehabilitative Medicine, the First Affiliated Hospital of Clinical Medicine of Guangdong Pharmaceutical University, Guangzhou, 510080 China

**Keywords:** Cerebral vascular disease, Sleep, Polysomnography, Sleep fragmentation, Markers

## Abstract

**Background:**

Sleep fragmentation was shown to be positively associated with cognitive impairment in patients with cerebral small vessel disease (CSVD); however, the underlying mechanisms are not well characterized. In this study, we sought to clarify this issue by investigating the relationship between non breathing-related sleep fragmentation and brain imaging markers in patients with CSVD.

**Methods:**

Eighty-four CSVD patients and 24 age- and sex-matched healthy controls were prospectively recruited. All subjects underwent 3.0 T superconducting magnetic resonance imaging and overnight polysomnography. Polysomnography parameters including sleep onset latency (SOL), total sleep time (TST); sleep efficiency (SE), wake after sleep onset (WASO), percentage of each sleep stage (N1, N2, N3 and rapid eye movement [REM]), arousal index (ArI), periodic limb movement in sleep index (PLSMI), and periodic limb movement related arousal index (PLMAI) were compared between CSVD patients and healthy controls. The relationship between arousal index and CSVD markers was explored in the CSVD group.

**Results:**

On polysomnography, CSVD patients showed significantly higher ArI, WASO, PLSMI, and PLMAI, and lower sleep efficiency and N^− 3^ ratio compared to healthy controls (*p* < 0.05). On ordinal logistic regression, higher ArI showed a positive association with the severity of periventricular white matter hyperintensity (odds ratio [OR] 1.121, 95% confidence interval [CI] 0.138–2.185) and perivascular space (OR 2.108, 95% CI 1.032–4.017) in CSVD patients, after adjusting for potential confounding variables.

**Conclusions:**

These preliminary results indicate that non breathing-related sleep fragmentation is common and related to the pathological markers of CSVD patients. Future prospective research is required to determine the causal relationship between sleep parameters and CSVD pathology.

## Background

The term cerebral small vessel disease (CSVD) refers to a series of clinical, imaging and pathological syndromes characterized by involvement of small arteries, arterioles, capillaries, and venules in the brain. The most common form is arteriolosclerosis which is also known as age-related and traditional vascular risk-factor-related small vessel disease [1]. The terminology used for imaging features of SVD and their definitions vary widely. Wardlaw et al. [2], an international working group from the Centres of Excellence in Neurodegeneration, completed a structured process to develop definitions and imaging standards for markers and consequences of CSVD. The neuroimaging features of CSVD include small subcortical infarcts, lacunes, white matter hyperintensities (WHMs), perivascular spaces (PVSs), cerebral microbleeds (CMBs), and brain atrophy.

Sleep fragmentation is characterized by recurrent and brief arousals that occur during nocturnal sleep. Elderly individuals are frequently affected by sleep fragmentation, which impairs the sleep quality [3]. Obstructive sleep apnea syndrome (OSAS) is a common cause of sleep fragmentation, and is also regarded as one of the non-traditional risk factors for cardiovascular and cerebrovascular diseases [4, 5]. However, OSAS is relatively uncommon compared to non-breathing related sleep complaints such as insomnia [6]. The estimated prevalence of insomnia symptoms ranges from 30 to 48% in the elderly while the estimated prevalence of insomnia disorder ranges from 12 to 20% [7]. To the best of our knowledge, there is limited data evaluating the status of non-breathing-related sleep fragmentation in CSVD patients; in addition, its relationship with the pathological changes in brain is not well characterized. Berezuk C (2015) assessed 26 patients with ischemic cerebrovascular disease using polysomnography (PSG) and magnetic resonance imaging (MRI); they observed a correlation of sleep efficiency (SE), wake after sleep onset (WASO) and N3 sleep duration with PVS in basal ganglia. However, patients with OSAS were not excluded from this study which may have confounded the results. Moreover, the small sample size in this study and absence of a control group reduce the significance of the study [8]. Other studies with larger samples have found an association between poor sleep quality [9] or long sleep duration [10] with white matter WMH in the brain; however, the subjects comprised of community-dwelling elderly individuals or stroke-free urban residents and not the patients; in addition, the sleep quality was evaluated only using subjective scales. Bella et al. [11] reported a linkage of white matter lesions with cognitive symptoms and poor outcomes of antidepressant therapy among elderly patients with subcortical ischemic vascular disease. In our previous study, we observed a positive association of sleep fragmentation with cognitive impairment in CSVD patients; however, we did not explore the underlying mechanisms, especially the relationship between sleep fragmentation and brain pathological changes. Moreover, the characteristics of sleep fragmentation could not be well understood due to the lack of healthy controls [12].

In the present study, we explored the relationship between objective non breathing-related sleep parameters and neuroimaging markers in a relatively larger sample of CSVD patients and a healthy control group. We hypothesized that non breathing-related sleep fragmentation is a common phenomenon in CSVD patients and is positively associated with specific CSVD imaging markers.

## Methods

### Design

This was a single-center cross-sectional case-control study. Consecutive patients with CSVD were recruited from the outpatient clinic of the Third Affiliated Hospital of Sun Yat-sen University between September 2017 and December 2018. Healthy spouses or siblings of the CSVD patients were enrolled in the healthy control group.

### Participants

A total of 84 CSVD patients were recruited based on the inclusion/exclusion criteria, along with 24 age- and sex-matched healthy controls. All participants underwent structured interviews and physical examination by a study physician who confirmed the diagnosis. Data pertaining to the following demographic and clinical characteristics were collected for all subjects: age; sex; body mass index (BMI); marital status; occupation; educational status; insomnia conditions (Table 1).

**Table 1:** Demographic and clinical characteristics of the study population

Groups	n	Age, mean (SD)	Male, n (%)	BMI ≥ 25, n (%)	Single, n (%)
Study group	84	60.1 ± 8.4	47 (56.0%)	37 (44.0%)	7 (8.3%)
Control group		61.8 ± 10.2	14 (58.3%)	11 (45.8%)	4 (16.7%)
*t/χ*^*2*^	24	0.136^a^	0.043^b^	0.024^b^	1.417^b^
*P*		0.728	0.836	0.877	0.234
**Groups**	**n**	**Retirement, n (%)**	**Education < 12 years, n (%)**	**Insomnia complaints, n (%)**	**Insomnia, n (%)**
Study group	84	38 (51.4%)	41 (48.8%)	63 (75.0%)	54 (64.3%)
Control group	24	11 (45.8%)	15 (62.5%)	9 (37.5%)	7 (29.2%)
*t/χ*^*2*^		0.221^b^	1.401^b^	11.813	9.366
*P*		0.638	0.236	0.001	0.002

^a^*t* value, ^b^ χ^2^ value

*Abbreviations*: *SD* standard deviation; *BMI* body mass index

#### Inclusion criteria

1. Age: 50–70 years.
2. Presence of at least one of the following cardiovascular risk factors: hypertension, atherosclerosis, diabetes mellitus, past or current smoking.
3. Presence of the following symptoms of CSVD: lacunar syndromes, cognitive, motor (gait), dysphagia, or mood disturbances. Subjects who were eligible because of a lacunar syndrome were included only > 3 months after the event to avoid acute effects on the outcomes.
4. Evidence of one or more MR markers of CSVD [2]: lacunars, WMHs with Fazekas grade 2 (early confluent) or higher [13], visible PVSs and CMBs.
5. Subjects in the control group had no cardiovascular risk factors, CSVD symptoms, or MR markers of CSVD.
6. Provision of written informed consent.

#### Exclusion criteria

1. Patients with larger subcortical or cerebral Water-shed infarctions (> 1.5 cm) on MR, as these are often embolic.
2. Patients with large artery disease - carotid, vertebral or intracranial stenosis > 50%;
3. Presence of severe mental disorders such as schizophrenia, bipolar disorder or major depression, alcohol and/or other drug abuse or dependence;
4. Presence of uncontrolled somatic disorders or sleep-disrupting comorbid medical conditions (e.g., moderate to severe rheumatoid arthritis); patients who required immediate psychiatric (e.g., imminently suicidal patients) or medical care (e.g., patients with acute cardiac symptoms)
5. Patients with breathing-related sleep disorders, restless legs syndrome, or circadian rhythm sleep disorders according to the criteria of the Diagnostic and Statistical Manual of Mental Disorders, Fifth Edition [14].
6. Apnea-hypopnea index ≥15 or/and periodic limb movement (PLM)-related arousal index ≥15 per hour of sleep during a screening laboratory polysomnogram;
7. Patients with CSVD caused by other reasons such as hereditary or inflammatory disorders or amyloidosis.
8. Patients who were being treated with medications that affect sleep within 2 weeks of initial screening. Medications that affect sleep include sleep-promoting medications (e.g., hypnotics/sedatives/neuroleptics/other novel agents) and wakefulness-promoting medications (e.g., modafinil, traditional stimulants).
9. Any inability to fulfill study data collection.

### Brain MRI scan

The General Electric 3 T superconducting MR scanner with 8 channels, head and neck coils was used for brain scanning. The acquisition sequences included 3D TOF-MRA, MRI T1Flair, T2WI, T2Flair and susceptibility weighted imaging (SWI). Images were rated by a certified and registered neuroradiologist in a blinded manner for the presence of the following four CSVD makers; the presence of each SVD makers was summed in an ordinal “SVD burden score” (range 0–4) [15].

Lacunes: rounded or ovoid lesions, > 3- and < 15-mm diameter, in the basal ganglia, internal capsule, centrum semiovale, or brainstem, exhibiting signal intensity of CSF on T2 and FLAIR, generally with a hyperintense rim on FLAIR, consistent with a previous acute small subcortical infarct or haemorrhage in the territory of one perforating arteriole. Presence of lacunes was defined as the presence of one or more lacunes (1 point if present).

WMHs: signal abnormality of variable size in the white matter that exhibits the following characteristics: hyperintensity on T2-weighted images such as fluid-attenuated inversion recovery, without cavitation (signal different from CSF). The severity of periventricular WMH (PWMH) and deep WMH (DWMH) were both coded according to the Fazekas scale from 0 to 3. Presence of WMH was defined as either (early) confluent DWMH (Fazekas score 2 or 3) or irregular PWMH extending into the deep white matter (Fazekas score 3) (1 point if present) [13].

PVSs: small (< 3 mm) punctate (if perpendicular) or linear (if longitudinal to the plane of scan) hyperintensities on T2 images in the basal ganglia. The severity of PVS was rated on a validated semiquantitative scale from 0 to 4 (0 = no PVS; 1 = < 10 PVS; 2 = 11–20 PVS; 3 = 21–40 PVS; and 4= > 40 PVS). The numbers refer to PVSs on one side of the brain; the higher score was used in case of asymmetry between the two sides. Presence of PVSs in the basal ganglia was counted if these were of moderate to severe (grade 2–4) (1 point if present) [16].

CMBs: small (generally 2–5 mm in diameter), homogeneous, round foci of low signal intensity on T2-weighted MRI or other sequences that are sensitive to susceptibility effects in the areas of cerebellum, brainstem, basal ganglia, white matter, or cortico-subcortical junction. Presence of CMBs was defined as the presence of any CMB (1 point if present).

### PSG

PSG was recorded in a sleep laboratory for 2 consecutive nights and the average value of each parameter obtained on the 2 consecutive nights was calculated. Recording of PSG started based on the subject’s usual sleeping habits; each patient’s sleep was recorded for a minimum of 8 h. The wireless telemetry polysomnography system (manufacturer: Germany SOMNOmedics; model: SOMNOscreen plus PSG +; analysis software “DOMINO”) was used to monitor sleep. Sleep recordings were scored by a psychologist who was blinded to the group identity. All PSG records were staged and scored based on the American Academy of Sleep Medicine Manual [17]. PSG measures reported included: sleep onset latency in minutes (SOL, the number of minutes it took for the participant to fall asleep after going to bed); total sleep time in minutes (TST, total minutes of sleep during the PSG recording); sleep efficiency % (SE %, total sleep time divided by time in bed); wake after sleep onset (WASO, the number of minutes being awake after initial sleep onset until last awakening); percentage of each sleep stage (N1, N2, N3 [slow wave sleep; SWS] and rapid eye movement [REM]); PLM in sleep index (PLMSI, the number of PLM/h); PLM arousal index (PLMAI, the number of PLM leading to arousal/h); apnea-hypopnea index (AHI, the mean number of apneas and hypopneas per hour of sleep. Score a respiratory event in adults as an apnea if both of the following are met: There is a drop in the peak signal excursion by ≥90% of pre-event baseline; The duration of the ≥90% drop in sensor signal is ≥10 s. Score a respiratory event as a hypopnea if all of the following are met: The peak signal excursions drop by ≥30% of pre-event baseline; The duration of the ≥30% drop in signal excursions is ≥10 s; There is ≥3% oxygen desaturation from pre-event base line or the event is associated with an arousal; arousal index (ArI, total number of episodes of arousal divided by the duration of sleep in hours. Electroencephalographic (EEG) arousal is defined as an abrupt shift in EEG frequency for 3–14 s, which may include theta, alpha and/or frequencies greater than 16 Hz but not spindles). The ArI average score of the subjects in the control group was used as the cut-off value to define higher ArI in CSVD patients.

Pittsburgh sleep quality index (PSQI)-Chinese Version: The PSQI is a self-reported questionnaire used to assess the quality of sleep over the past one month. It contains seven domains including subjective sleep quality, sleep latency, sleep duration, sleep efficiency, and sleep disturbances, use of sleeping pills and daytime dysfunction. Each domain is scored on a scale of 0 to 3. The global PSQI score ranges from 0 to 21; a higher score indicates poorer sleep quality [18].

### Statistical methods

SPSS software package (version 20.0) was used for statistical analyses. Descriptive statistics are presented as mean ± standard deviation for normally-distributed continuous variables and as frequencies or proportions for categorical variables. The student’s *t* test was used to assess the between-group differences with respect to sleep parameters. Logistic regression was performed to evaluate the association between sleep fragmentation and CSVD imaging markers. Two-sided *P*-values ≤0.05 were considered indicative of statistical significance.

## Results

### Clinical characteristics of subjects

A total of 121 consecutive patients with CSVD were screened for recruitment. Of these, 37 patients were excluded based on the following patient-selection criteria: presence of OSAS (*n* = 11); presence of other medical conditions that interfere with sleep (*n* = 2); patients taking sleep pills (*n* = 12); (4) presence of major depression (*n* = 4); refusal to participate (*n* = 8). A total of 84 patients and 24 healthy controls were finally enrolled in the study. There were no significant between-group differences with respect to demographic characteristics (*p* > 0.05) (Table 1). Among the 84 CSVD patients, 63 complained about poor sleep quality and 54 patients qualified the DSM-5 criteria for chronic insomnia.

### PSG parameters

Subjects in the study group showed significantly lower SE and N-3 ratio compared to the control group; the WASO, N-1 ratio, ArI, PLMSI, and PLMAI of the study group were significantly higher than that in control group (*p* < 0.05). These findings indicated that CSVD patients had more severe objective sleep fragmentation than normal controls, especially during the slow wave sleep (Table 2).

**Table 2:** Comparison of PSG measures

Groups	n	SOL, min	TST, min	SE, %	WASO, min	AHI, per hour	PLMSI
Study group	84	25.3 ± 14.5	314.8 ± 88.5	61.5 ± 10.8	131.5 ± 51.2	3.4 ± 1.5	10.3 ± 8.7
Control group		19.8 ± 13.8	368.6 ± 98.2	72.4 ± 13.4	96.8 ± 42.1	3.8 ± 1.2	6.7 ± 5.9
*t*	24	1.723	1.328	2.397	2.759	0.124	2.605
*P*		0.095	0.195	0.031	0.011	0.957	0.019
**Groups**	**n**	**REM (%)**	**NREM-1 (%)**	**NREM-2 (%)**	**NREM-3 (%)**	**ArI, per hour**	**PLMAI**
Study group	84	16.4 ± 4.7	19.2 ± 3.4	52.2 ± 11.4	10.4 ± 4.5	26.9 ± 7.4	5.6 ± 5.2
Control group	24	19.5 ± 5.5	12.1 ± 1.2	53.4 ± 9.5	13.5 ± 5.2	19.8 ± 7.7	3.7 ± 2.8
*t*		2.104	4.241	0.285	2.214	2.472	2.352
*P*		0.045	0.000	0.657	0.039	0.021	0.036

*Abbreviations*: *SOL* sleep onset latency; *TST* total sleep time; *SE* sleep efficiency; *WASO* wake after sleep onset; *REM* rapid eye movement sleep; *NREM* non rapid eye movement sleep; *AHI* apnea–hypopnea index; *ArI* arousal index; *PLMSI* periodic limb movement in sleep index; *PLMAI* periodic limb movement related arousal index

### PSQI measures

Participants in the study group had significantly lower SE, worse daytime dysfunction, and higher PSQI total score than the control group (*p* < 0.05). These findings indicated that patients with CSVD had worse subject sleep quality than normal controls (Table 3).

**Table 3:** Comparison of PSQI measures

Groups	N	Total score	Sleep quality	Sleep latency	Sleep time
Study group	84	9.3 ± 5.2	1.5 ± 1.0	1.3 ± 1.2	1.7 ± 1.1
Control group	24	5.9 ± 2.1	0.9 ± 0.4	0.8 ± 0.6	1.2 ± 0.7
*t*		2.584	1.816	1.931	0.834
*p*		0.015	0.084	0.062	0.416
**Groups**	**N**	**Sleep efficiency, %**	**Sleep disturbances**	**Medication use**	**Daytime dysfunction**
Study group	84	65.3 ± 13.4	1.7 ± 0.6	–	1.5 ± 0.8
Control group	24	76.2 ± 8.2	1.0 ± 0.3	–	1.1 ± 0.3
*t*		2.389	1.682	–	2.392
*p*		0.032	0.104	–	0.030

### Imaging markers in CSVD patients with different level of ArI

Among the 84 CSVD patients, 23 (27.4%) patients had lacuna, 25 (29.8%) had CMB, 42 (50%) had WMH (DWMH Fazekas 2–3 and/or PWMH Fazekas 3), and 40 (47.6%) had PVS. There was no significant difference between patients with higher and lower ArI with respect to age, sex, education level, BMI, or vascular risk factors (*p* > 0.05). Patients with higher ArI showed significantly more severe PWMH and PVS than those with lower ArI (*p* < 0.05) (Table 4).

**Table 4:** Comparisons of CSVD markers in patients with different ArI level

Groups	n	CSVD burden	Lacuna presence	CMB presence	WMH presence	PWMH severity	DWMH severity	PVS severity	PVS presence
Higher ArI	53	1.7 ± 0.7	15 (28.3%)	18 (34.0%)	27 (50.9%)	1.9 ± 0.9	1.4 ± 1.0	2.4 ± 1.1	28 (52.8%)
Lower ArI	31	1.5 ± 0.7	8 (25.8%)	7 (22.6%)	15 (48.4%)	1.3 ± 1.0	1.6 ± 1.1	1.6 ± 0.9	15 (48.4%)
*t/χ*^*2*^		0.123^a^	0.061^b^	1.212^b^	0.051^b^	2.172^a^	0.723^a^	2.426^a^	0.155^b^
*P*		0.911	0.805	0.271	0.821	0.043	0.475	0.026	0.694

*Abbreviations*: *CSVD* cerebral small vessel disease; *CMB* cerebral microbleed; *WMH* white matter hyperintensities; *PWMH* periventricular *WMH* DWMH deep *WMH* PVS, perivascular space

^a^*t* value, ^b^ χ^2^ value

### Relationship between sleep fragmentation and CSVD imaging markers regression

Ordinal logistic regression was performed to evaluate the association between sleep fragmentation and CSVD markers of patients in the study group. PWMH severity and PVS severity were the outcomes; ArI (≥19.8/h or < 19.8/h) was the exposure; and potential confounders [TST (≥6 h or < 6 h), WASO (≥30 min or < 30 min), SE (≥85% or < 85%), N-3 sleep ratio, PLMSI, and PLMAI] were the independent variables. The results showed that higher ArI was significantly associated with PWMH severity (odds ratio [OR] 1.121, 95% confidence interval [CI] 0.138–2.185) and PVS severity (OR 2.108, 95% CI 1.032–4.017), after adjusting for all independent variables. These findings indicated that sleep fragmentation may aggravate pathological process of CSVD (Table 5).

**Table 5:** Results of ordinal logistic regression showing the relationship of sleep parameters and CSVD imaging markers

Variables	ArI	TST	WASO	SE	%, N-3	PLMSI	PLMAI
PWMH severity							
OR	1.121	0.754	2.835	0.825	0.756	0.703	0.613
95% CI	0.138–2.485	0.369–2.151	0.728–4.685	0.367–2.010	0.235–1.423	0.181–2.733	0.143–2.632
*P*	0.034	0.637	0.152	0.670	0.431	0.611	0.511
PVS severity							
OR	2.108	0.964	1.428	0.965	1.052	1.185	1.241
95% CI	1.032–4.017	0.204–2.258	0.439–2.284	0.312–1.941	0.534–2.294	0.982–1.430	0.909–1.532
*P*	0.027	0.568	0.339	0.785	0.654	0.086	0.255

*Abbreviations*: *CSVD* cerebral small vessel disease; *PWMH* periventricular white matter hyperintensities; *PVS* perivascular space; *ArI* arousal index; *TST* total sleep time; *WASO* wake after sleep onset; *SE* sleep efficiency; *N-3* non rapid eye movement sleep-stage 3; *OR* odds ratio; *CI* confidence interval; *PLMSI* periodic limb movement in sleep index; *PLMAI* periodic limb movement related arousal index

## Discussion

The first finding of this study was that the proportion of patients with non-breathing-related sleep disorders among CSVD patients was much higher than that of patients with breathing-related sleep disorders. Of the 121 CSVD patients screened, 11 (9.1%) qualified the OSAS diagnostic criteria, 63 (52.1%) had complaints of insomnia, and 54 (44.6%) qualified the criteria for chronic insomnia. This proportion is also much higher than that among elderly subjects in previous studies [7], and also higher than that of chronic insomnia (29.2%) in the healthy controls in this study. This indicates high co-morbidity of non-breathing-sleep disorders and CSVD.

The second finding of this study was that the severity of non-breathing-sleep fragmentation in CSVD patients was significantly higher than that in healthy controls. In the elderly, along with the reduced homeostatic sleep pressure and reduced circadian signals, the following age-related changes in sleep have been observed: decreased total sleep time, sleep efficiency, and slow-wave sleep; and increased waking after sleep onset [19]. In this study, there was no significant difference between CSVD patients and healthy controls with respect to total sleep time; however, the ArI and WASO were significantly higher than that in the control group. This indicated that disruption of sleep continuity was more serious in CSVD patients. Accordingly, worse sleep efficiency, lower radio of N-3 and REM sleep, and greater impairment of daytime function was observed in CSVD patients. It is well known that CSVD often causes cognitive impairment; this impairment was found to be aggravated by decrease in N3 and REM sleep in our previous study [12]. In a recent study by Perrault et al. [20], compared to a stationary position, continuous rocking of patients during night was found to shorten the latency to NREM sleep and prolong sleep continuity, as indexed by increased N3 duration and fewer arousals. Therefore, reducing non breathing-related sleep fragmentation, such as by continuous rocking during sleep, could be a new therapeutic target for cognitive impairment of CSVD patients.

The third finding of this study was the positive correlation of the degree of sleep fragmentation with the severity of PWMH and PVS in CSVD patients. PVS are the extension of subarachnoid spaces that surround the smaller arteries and veins in brain. These are interstitial fluid-filled spaces which act as a glymphatic system and play an important role in interstitial clearance [21]. Intriguingly, the glymphatic system functions mainly during sleep and is largely disengaged during wakefulness. Using real-time two-photon microscopy in live mice, Xie (2013) found that natural sleep or anesthesia are associated with a 60% enlargement in the interstitial space, resulting in a significant increase in convective exchange of cerebrospinal fluid with interstitial fluid and an increase in the rate of clearance of β-amyloid and other metabolites during sleep [22]. However, humans and mice may not have the same glymphatic drainage system in brain [23]. Our study supported the hypothesis that sleep may promote the clearance of metabolites in the human brain through perivascular channels. In this study, PVS showed a positive correlation with sleep fragmentation, which indicated that disruption of sleep continuity may be associated with inefficiency and blockage of perivascular drainage, potentially resulting in enlargement of the perivascular space.

WMH of presumed vascular origin are very common in elderly and regarded as typical markers of CSVD. Due to limited pathological studies, the underlying pathology of WMH is not well characterized and is considered rather heterogeneous, the heterogeneity reflecting different disease stages. Possible mechanisms include incomplete infarct, chronic hypoperfusion, immune and inflammatory activation, and chronic edema due to increased permeability of the blood brain barrier [24]. The mechanism linking WMHs with non breathing-related sleep fragmentation is not clear; however, several studies have shown that sleep disturbances may be related to pathological progress of WMH via the following mechanisms: by decreasing the exchange of cerebrospinal fluid with interstitial fluid and the clearance of metabolities such as β-amyloid [22], promoting neuroinflammation [25], generating new molecules such as free radicals [26], increasing the permeability of the BBB [27], inducing pulse pressure surges [28], and increasing vascular stiffness and altering cerebral perfusion [29]. In the present study, subjects with a higher ArI showed more severe PWMHs, which supports the hypothesis that non breathing-related sleep fragmentation could be related to the pathological mechanism of WMHs. In the present study, the degree of sleep fragmentation in CSVD patients showed a positive correlation with the severity of PWMH and PVS; as mentioned above, the pathologic progress of WMH and PVS might be related to clearance of β-amyloid and other metabolites, production of neuroinflammatory molecules, and oxidative stress. Since WMH and PVS were regarded as typical markers of CSVD, the above factors may represent the neurochemical link between sleep fragmentation and cerebrovascular disease and may be related to the pathologic progress of WMH and PVS.

The strengths of our study include the evaluation of a group of clinical patients using objective measures of sleep and CSVD markers by magnetic resonance imaging. However, the cross-sectional design does not permit any causal inferences.

## Conclusion

In conclusion, we found an association between non breathing-related sleep fragmentation and increased severity of PWMH and PVS. Prospective studies are required to explore the causal relationship of disturbed sleep and the pathological markers of CSVD.

## Data Availability

The data sets generated and analyzed during the current study are not available publicly as ethical clearance was not obtained to share data publicly. However, The data is available from the corresponding author on reasonable request.

## Abbreviations

CMBs: Cerebral microbleeds
CSVD: Cerebral small vessel disease
MRI: Magnetic resonance imaging
OSAS: Obstructive sleep apnea syndrome
PLMAI: Periodic limb movement related arousal index
PLMSI: Periodic limb movement in sleep index
PSG: Polysomnography
PSQI: Pittsburgh sleep quality index
PVS: Perivascular space
SE: Sleep efficiency
WASO: Wake after sleep onset
WMH: White matter hyperintensity
